# Diagnoses and treatments for participants with interstitial lung abnormalities detected in the Yorkshire Lung Screening Trial

**DOI:** 10.1136/bmjresp-2022-001490

**Published:** 2023-08-23

**Authors:** Sara Upperton, Paul Beirne, Bobby Bhartia, Alison Boland, Claire Bradley, Philip A J Crosbie, Mike Darby, Claire Eckert, Rhian Gabe, Neil Hancock, Martyn P T Kennedy, Jason Lindop, Suzanne Rogerson, Bethany Shinkins, Irene Simmonds, Tim J T Sutherland, Matthew E J Callister

**Affiliations:** 1 Department of Respiratory Medicine, Leeds Teaching Hospitals NHS Trust, Leeds, UK; 2 Department of Radiology, Leeds Teaching Hospitals NHS Trust, Leeds, UK; 3 Craigavon Area Hospital, Southern Health and Social Care Trust, Portadown, UK; 4 Division of Infection, Immunity & Respiratory Medicine, The University of Manchester, Manchester, UK; 5 Leeds Diagnosis and Screening Unit, University of Leeds, Leeds, UK; 6 Wolfson Institute of Population Health, Queen Mary University of London, London, UK; 7 Department of Research and Innovation, Leeds Teaching Hospitals Trust, Leeds, UK

**Keywords:** interstitial fibrosis, lung cancer, imaging/CT MRI etc

## Abstract

**Introduction:**

Interstitial lung abnormalities (ILA) are relatively common incidental findings in participants undergoing low-dose CT screening for lung cancer. Some ILA are transient and inconsequential, but others represent interstitial lung disease (ILD). Lung cancer screening therefore offers the opportunity of earlier diagnosis and treatment of ILD for some screening participants.

**Methods:**

The prevalence of ILA in participants in the baseline screening round of the Yorkshire Lung Screening Trial is reported, along with the proportion referred to a regional ILD service, eventual diagnoses, outcomes and treatments.

**Results:**

Of 6650 participants undergoing screening, ILA were reported in 169 (2.5%) participants. Following review in a screening review meeting, 56 participants were referred to the ILD service for further evaluation (0.8% of all screening participants). 2 participants declined referral, 1 is currently awaiting review and the remaining 53 were confirmed as having ILD. Eventual diagnoses were idiopathic pulmonary fibrosis (n=14), respiratory bronchiolitis ILD (n=4), chronic hypersensitivity pneumonitis (n=2), connective tissue disease/rheumatoid arthritis-related ILD (n=4), asbestosis (n=1), idiopathic non-specific interstitial pneumonia (n=1), sarcoidosis (n=1) and pleuroparenchymal fibroelastosis (n=1). Twenty five patients had unclassifiable idiopathic interstitial pneumonia. Overall, 10 people received pharmacotherapy (7 antifibrotics and 3 prednisolone) representing 18% of those referred to the ILD service and 0.15% of those undergoing screening. 32 people remain under surveillance in the ILD service, some of whom may require treatment in future.

**Discussion:**

Lung cancer screening detects clinically significant cases of ILD allowing early commencement of disease-modifying treatment in a proportion of participants. This is the largest screening cohort to report eventual diagnoses and treatments and provides an estimate of the level of clinical activity to be expected by ILD services as lung cancer screening is implemented. Further research is needed to clarify the optimal management of screen-detected ILD.

**Trial registration number:**

ISRCTN42704678.

**WHAT IS ALREADY KNOWN ON THIS TOPIC**

- Interstitial lung abnormalities are common findings on low-dose CT screening scans performed in lung cancer screening programmes, and in some cases may represent interstitial lung disease (ILD) for which effective pharmacotherapy is now available.

**WHAT THIS STUDY ADDS**

- Few previous studies have reported the eventual diagnoses and treatments of cases of screen-detected ILD; here, we report the largest lung cancer screening cohort to include eventual ILD diagnoses and treatments. Overall, 0.8% of participants undergoing baseline screening were diagnosed with ILD and 0.15% have commenced treatment to date.

**HOW THIS STUDY MIGHT AFFECT RESEARCH, PRACTICE OR POLICY**

- This study shows that lung cancer screening detects clinically significant cases of ILD fulfilling criteria for pharmacotherapy. The proportions of participants needing referral to ILD services and requiring eventual treatment reported here will help capacity planning for services alongside future roll-out of lung cancer screening programmes.

## Introduction

Low-dose CT (LDCT) screening for lung cancer has been shown to reduce lung cancer-specific and all-cause mortality in the National Lung Screening Trial (NLST)^1^ and screening was introduced in the USA in 2013.^2^ A second randomised trial, the Nederlands–Leuvens Longkanker Screenings Onderzoek (NELSON) study,^3^ reported in 2020 confirming a reduction in lung cancer mortality. As a result, many nations are now considering implementing screening, and in September 2022, the UK National Screening Committee issued a recommendation for a nationwide-targeted lung cancer screening programme.^4^ Alongside the anticipated rise in activity relating to cancer diagnoses and treatments, screening implementation will also result in additional incidental findings on LDCT scans.^5^ For some screening participants, detection of these incidental findings will allow early treatment of other non-malignant conditions, thereby possibly augmenting the clinical benefit of LDCT screening. However, the impact of this additional clinical activity on related services needs to be considered as part of implementation planning.

The term interstitial lung abnormalities (ILA) refers to a variety of incidental radiological findings on CT imaging, some of which are transient and inconsequential,^6^ but some of which could represent interstitial lung disease (ILD), as described in a recent position paper from the Fleischner Society.^7^ ILA are associated with both age^8^ and smoking history,^9^ both key eligibility criteria for lung cancer screening. Unsurprisingly therefore, ILA are common incidental findings on LDCT screening scans, with a reported prevalence of 20% in a retrospective analysis of participants in NLST,^10^ although prevalence reported in other screening trials and programmes has been somewhat lower at around 8%–10%.^6 11 12^ A proportion of patients with ILA may progress to clinically significant ILD, for which there are a number of therapies with proven efficacy.^13–15^ Therefore, LDCT screening for lung cancer could provide an opportunity to diagnose and treat patients with ILD at an earlier stage.

Many screening participants with ILA can simply be kept under radiological surveillance, usually as part of the ongoing screening programme. However, a proportion of participants will have more significant radiological abnormalities meriting referral to an ILD service for further evaluation and, if appropriate, pharmacotherapy. Although many studies have reported the diagnostic prevalence of ILA,^6 10–12 16^ few have assessed the downstream impact, specifically the proportion of participants who need referral to specialist services and who end up receiving pharmacological treatment for their screen-detected ILD. Here, we report the eventual diagnoses and treatments for participants referred to an ILD service following the baseline round of screening as part of the Yorkshire Lung Screening Trial (YLST).

## Methods

### YLST study design

The full protocol of YLST has been published previously.^17^ Briefly, people aged 55–80 years who had ever smoked were invited to a telephone-based risk assessment for lung cancer. Those at higher risk of lung cancer according to any of three criteria used (US Preventive Services Task Force criteria)^2^; PLCO_M2012_ (Prostate, Lung, Colorectal and Ovarian cancer screening trial model)≥1.51% over 6 years^18^; LLP_v2_ (Liverpool Lung Project)≥5% over 5 years^19^ were invited for a Lung Health Check comprising a LDCT scan, demographic and clinical questionnaires, spirometry and a colocated opt-out smoking cessation service. Baseline recruitment to YLST took place between November 2018 and February 2021.

### Patient and public involvement (PPI)

Patients and members of the public were closely involved in the YLST design. A group of four PPI representatives met regularly during the study design period, providing feedback on the design and suggesting amendments which were incorporated. PPI feedback was presented to both the Research Ethics Committee and Confidentiality Advisory Group. In addition, separate PPI sessions were involved using a pre-existing patient support group, the Leeds Lung Cancer and Mesothelioma Patient Support Group comprising patients and family members. Four events were held during the trial design phase where plans were presented to the group for feedback and comment.

### Participant history and symptoms

At the LHC, participants were asked about a history of respiratory disease, presence of respiratory symptoms (exertional breathlessness, chronic cough, regular sputum production, wheeze, frequent winter bronchitis), number of hospital admissions for chest problems, number of antibiotic courses in the last 12 months, modified Medical Research Council (mMRC) dyspnoea score (0–4), and WHO performance status (0–4). Smoking status was recorded and number of pack years calculated. Participants had exhaled carbon monoxide measured and performed routine spirometry up to the onset of the COVID-19 pandemic in March 2020; spirometry did not continue following recommencement of screening in July 2020.

### LDCT scan reporting and review

LDCT scans for YLST are reported by a team of eight consultant radiologists with a subspecialty interest in thoracic imaging. Scans are reported using Veolity (MeVis AG), a bespoke software package for Lung Cancer screening including automated volumetry and computer-aided detection. In addition to protocolised reporting of nodules and possible cancers, free-text comments allow reporting of other incidental abnormalities with the option of a flag for clinically significant findings requiring clinical review. All scans with such a flag are reviewed at a screening review meeting (SRM) attended by a consultant thoracic radiologist and a consultant respiratory physician. At the SRM, electronic healthcare records and previous imaging performed outside of YLST are available for review. The radiologists who attend the SRMs also regularly contribute to ILD multidisciplinary team (MDT) meetings. Scans without a clinical flag are signed off by a respiratory physician, with the option of listing for SRM if further discussion is thought necessary.

The YLST reporting radiologists were asked to mention ILA in their clinical report in all cases, and to mark the scan report as having a significant incidental finding if there were clinically significant ILA defined as evidence of traction bronchiectasis or >10% reticulation. Some cases that were not flagged but had comments regarding ILA were also discussed at the discretion of YLST physicians. Following discussion at an SRM, if a clinically significant previously undiagnosed ILD was thought possible, a telephone clinic appointment with a YLST clinician was arranged. At this review, further relevant history was obtained, participants were advised of the scan findings and onward referral to the regional ILD service was organised. A letter outlining the LDCT findings and any further management was sent to both the patient and general practitioner.

### Data collection

All participants with ILA discussed at an SRM were prospectively logged, including the outcome of the telephone consultation. In order to identify ILA that did not meet the predefined criteria for clinically significant disease, a search was performed on a dataset of all baseline LDCT scans for terms in the comments or SRM sections relating to ILA. Search terms included: interstitial, fibrosis, honeycombing, interstitial lung abnormality, ILD, idiopathic pulmonary fibrosis, usual interstitial pneumonia, non-specific interstitial pneumonia, cryptogenic organising pneumonia, bronchiolitis obliterans organising pneumonia, desquamative interstitial pneumonia, respiratory bronchiolitis ILD (RB-ILD) and acronyms thereof. Electronic healthcare records were then retrospectively reviewed for all cases and clinical outcomes recorded, including whether participants were referred to the regional ILD service, outcomes within the ILD service (specifically diagnosis from the ILD MDT, pulmonary function test monitoring, initiation of treatment for ILD, discharge from the service), lung cancer diagnosis, palliative care referral and death.

### Statistical analysis

Statistical analysis was performed using GraphPad Prism, with comparison between those with ILD (known and newly referred) and those without ILD using methodologically appropriate tests. YLST is registered with the ISRCTN (reference ISRCTN42704678).

## Results

Baseline LDCT screening scans were performed on 6650 participants during the prevalent round of screening between November 2018 and February 2021. ILA were reported in 169 cases (2.5%) of which 153 cases were discussed in the SRM, usually because these cases were flagged as clinically significant, but occasionally at the discretion of the respiratory physician signing off the case.

Of the 153 patients discussed in the SRM, 10 were known to the ILD service and no further action was needed and 87 were deemed to be not clinically significant and not referred. Thus, 56 patients (0.8% of the population undergoing prevalence LDCT screening) were identified as needing referral to the ILD service for initial discussion in the MDT meeting and subsequent review in the outpatient clinic. Demographic and clinical information is shown in table 1 describing the characteristics of those participants with ILD (known n=10, newly referred n=56) and those without ILD (n=6584). Participants with ILD were older than those without ILD (mean±SD 72.6±6.3 years vs 68.4±7.0, p<0.0001) and more likely to be male (76% vs 55%, respectively, p=0.001). There were no significant differences between these two groups according to Index of Multiple Deprivation, mMRC dyspnoea score or WHO performance status. The proportion of participants still smoking was similar between those with and without ILD (30% vs 35%, p=0.491) as were participant smoking histories. Lung function parameters (forced expiratory volume in one second (FEV1), FEV_1_ % predicted, forced vital capacity (FVC), FVC % predicted) were similar between the two groups.

**Table 1:** Demographic and clinical information regarding participants with and without interstitial lung disease

	Interstitial lung disease (known or newly diagnosed)		No interstitial lung disease		Comparison (p value)
Number of participants, % of overall eligible cohort	66	1%	6584	99%	
Age, years, mean, SD	72.6	6.3	68.4	7.0	<0.0001
Male, n, %	50	76%	3618	55%	0.001
IMD rank, median, IQR (lower=more deprived)	13 838	3907–21 622	3605–21 916		0.885
mMRC dyspnoea score, n, %					0.435
0–1	51	77%	5384	82%	
2–4	15	23%	1200	18%	
WHO performance status, n, %					0.108
0–1	56	85%	6008	91%	
2–3	10	15%	576	9%	
Smoking status, n, %					0.491
Current (within the last month)	20	30%	2313	35%	
Ex-smoker	46	70%	4271	65%	
Smoking history (pack years), median, IQR	32	22–47	35	26–45	0.447
Spirometry, mean, SD					
FEV_1_ (L)	2.2	0.7	2.2	0.7	0.642
FEV_1_ % predicted	93	26	89	21	0.317
FVC (L)	3.1	0.9	3.2	0.9	0.569
FVC % predicted	99	23	103	20	0.259
Screen-detected lung cancer, n, %	5	7.5%	158	2.4%	0.015

FEV_1_, forced expiratory volume in one second; FVC, forced vital capacity; IMD, Index of Multiple Deprivation; mMRC, modified Medical Research Council.

**Figure 1:**
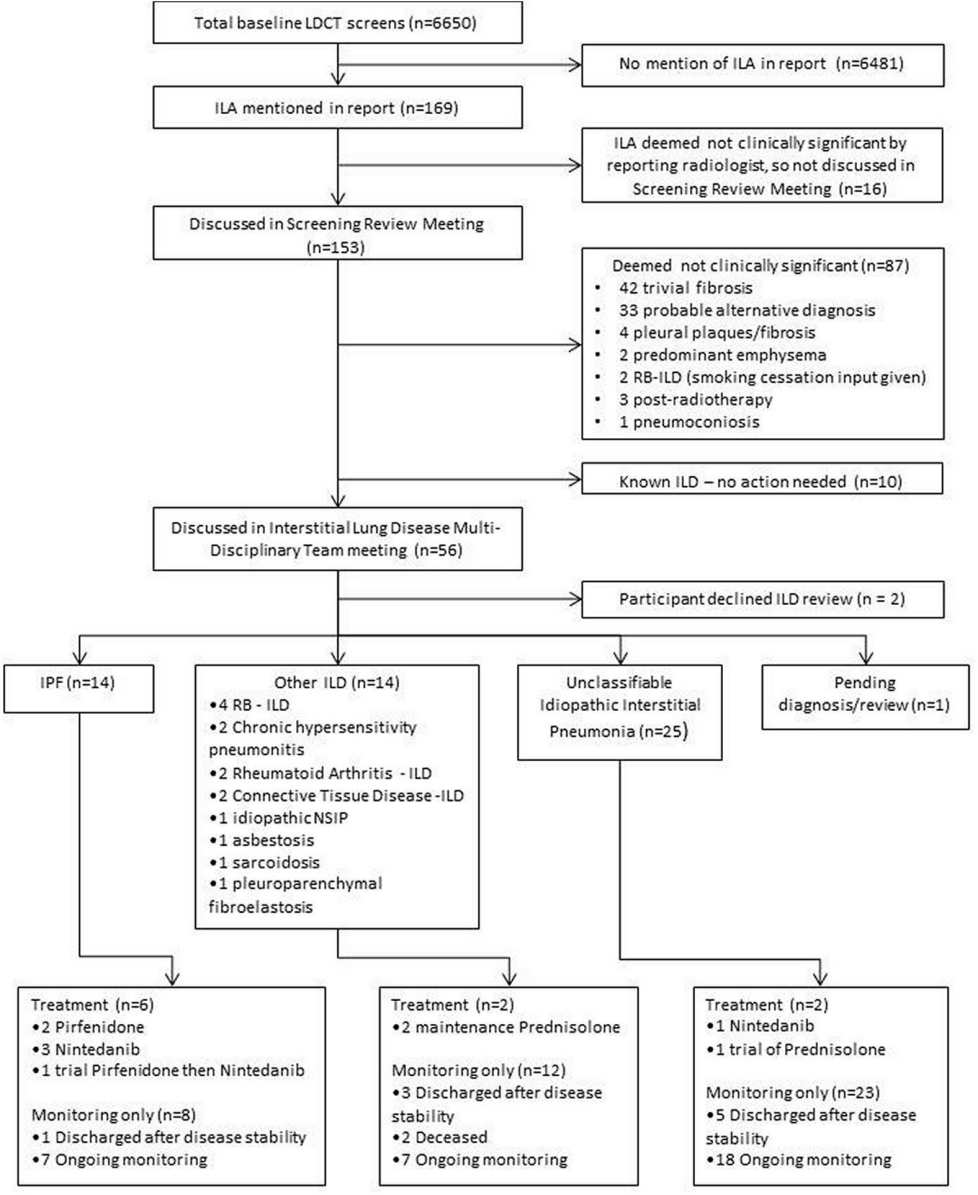
Flowchart for eventual diagnosis and treatment for participants with interstitial lung abnormalities reported on low-dose screening CT scans. ILA, interstitial lung abnormality; IPF, idiopathic pulmonary fibrosis; LDCT, low-dose CT; NSIP, non-specific interstitial pneumonia; RB-ILD, respiratory bronchiolitis interstitial lung disease.

Two patients refused referral to the ILD clinic, and one patient is awaiting clinic review and a final diagnosis under the ILD team. A total of 53 patients have therefore received a final diagnosis and a consort diagram of diagnoses and treatments is shown in figure 1. The most common single-specific diagnosis was idiopathic pulmonary fibrosis, found in 14 patients (26% of referred cases with a final diagnosis). Of these patients, 6 had an FVC less than 80% following measurement of full pulmonary function tests, thus meeting UK National Institute for Health and Care Excellence (NICE) criteria for antifibrotic treatment (2 pirfenidone, 3 nintedanib, 1 sequential treatments of pirfenidone then nintedanib).^20 21^ Two patients were diagnosed with chronic hypersensitivity pneumonitis, one of whom was started on maintenance prednisolone. Two patients were diagnosed with rheumatoid arthritis associated ILD one of whom commenced maintenance prednisolone. A total of 25 patients were diagnosed with unclassifiable idiopathic interstitial pneumonia. Of these, one subsequently received a course of prednisolone for a presumed pneumonitis flare, and one showed progressive fibrotic change on subsequent CT and was treated with nintedanib according to NICE guidelines.^22^

Thus, of 53 patients seen by the ILD service with a final diagnosis, 10 received pharmacotherapy (7 patients with antifibrotics, 3 patients with prednisolone) comprising 18% of those referred and 0.15% of all those undergoing prevalence LDCT screening. A total of 32 patients remain under active monitoring in the service with ongoing surveillance of lung function, 9 patients have now been discharged from the service after a period of stability and 2 patients died of unrelated causes.

The most common reason for non-referral of cases to the ILD service was the trivial or mild nature of the ILA, as assessed by either the initial reporting radiologist or the radiologist reviewing the case in the SRM (n=42). In 33 cases, review in the SRM concluded that the LDCT findings were due to alternative causes (inflammatory change, infection or heart failure) rather than true ILA. Two cases were thought to represent classic RB-ILD but were not referred, as the patients had already engaged with the smoking cessation team on the mobile unit. In other cases, the reported ILA were thought to represent postradiation fibrosis, pleural fibrosis, pneumoconiosis or predominant emphysema, and thus patients were not referred to the service.

Considering lung cancer diagnoses in the screening programme to date, a higher proportion of participants with ILD have been diagnosed with cancer compared with those without ILD (7.5% vs 2.4%, respectively, OR 3.16, 95% CI 1.25 to 7.94, p=0.015). Of the 103 participants with ILA mentioned in their LDCT reports who were not referred to the ILD service, 5 (4.9%) were subsequently diagnosed with lung cancer, thus considering the whole cohort with ILA/ILD 10 out of 169 participants (5.9%) were diagnosed with lung cancer.

## Discussion

In this analysis of ILA reported in a prevalence LDCT screening round, 2.5% of cases had mention of ILA in their CT report and 0.8% of cases were deemed clinically significant needing referral to the ILD service. Of those cases referred, IPF was the most common specific diagnosis (14 cases comprising 26% of referred cases with a final diagnosis), with 25 patients having unclassifiable idiopathic interstitial pneumonia (comprising 47% of referred cases with a final diagnosis). In total, 10 patients received pharmacotherapy (7 with antifibrotics and 3 with prednisolone) comprising 18% of all referred cases and 0.15% of all screening participants.

In our cohort, participants with ILD were older than those without ILD, in keeping with the previous reports.^8 10^ Similarly, higher rates of interstitial changes in men compared with women have been reported in other screening series.^6^ Additionally, the higher rate of lung cancer diagnosis in participants with ILD and ILA was reported in the NLST^10^ and other studies.^23^

The prevalence of ILA reported in this series is lower than that reported elsewhere (2.5% here compared with 8%–10% in other screening series^6 11 12^ and 20% in a retrospective review of cases from the NLST).^10^ A recent case series from another UK lung screening programme reported ILA in 4.2% of a screening cohort following 2 rounds of LDCT screening.^24^ In this study, some cases of ILA were newly detected on the incident (second) scan. This may in part explain why the prevalence of ILA was higher than in the current study, which only includes the baseline round of screening. Reporting radiologists in YLST were asked to comment on all cases of ILA for research purposes, although the reporting protocol indicated that a flag for review in the SRM should only be used for clinically significant cases defined by traction bronchiectasis or >10% reticulation. It is possible that radiologists did not follow the instruction to record cases of milder ILA not fulfilling the criteria for clinical significance, leading to this lower-reported prevalence. In addition, the 10% threshold for reticulation used in this study is higher than that described in the Fleischner Society statement^7^ and other guidance^25^ and may have contributed to lower prevalence and referral rates.

The current study is the largest to date to report eventual ILD diagnoses and the subsequent clinical outcomes following assessment. Two smaller studies from the UK have reported very different prevalence rates for new diagnoses of ILD across two rounds of LDCT screening. Only 1 study noted 28 new ILD diagnoses from 1853 screening participants (1.5%) with 0.6% commenced on pharmacotherapy.^24^ Another study reported only 3 diagnoses of ILD from 1409 screening participants (0.2%); no information was provided regarding treatment.^5^ The current study reported 53 new ILD diagnoses from 6650 screening participants (0.8%), although this was only from a baseline round of screening. All participants in YLST are invited for a second round of screening after a 2-year interval, and in due course, it will be possible to assess radiological progression in the 103 patients with ILA mentioned in their LDCT screening report but not referred to the ILD service. In addition, ‘interval ILD diagnoses’ whereby participants not referred to the ILD service are subsequently diagnosed outside the screening programme in the interscreen interval will be assessed.

Other studies that have assessed changes in ILA over a 2-year interval reported 33% improving, 47% remaining stable and 20% progressing.^6^ The Fleischner Society position paper recommended risk factor reduction for all participants with ILA.^7^ Overall, 30% of those participants found to have ILA on their screening LDCT in YLST were currently smoking, and this is therefore likely to represent the most significant risk factor in the context of screening. All participants in the YLST were offered an immediate opt-out colocated consultation with a smoking cessation practitioner irrespective of their screening result, and the overwhelming majority took up this offer.^17 26^

### Strengths and limitations

This is the largest study to date to describe downstream impacts of screen-detected ILA including the proportion of screening participants needing review in an ILD service and the proportion eventually receiving pharmacotherapy. The main limitation is that findings are only available from the baseline round of screening (the first incidence screening round is still underway). The proportions of participants needing review by the ILD service, and fulfilling criteria for pharmacotherapy will therefore increase with time—first as people already under surveillance with the ILD team experience a decline in lung function parameters and therefore cross treatment thresholds and second as incident screening rounds reveal new onset or progression of ILA compared with the baseline screen.

### Summary

Earlier detection of ILD through lung cancer screening has the potential to offer clinical benefit compared with symptomatic presentation, by facilitating earlier commencement of pharmacotherapeutic agents with proven efficacy. This study has confirmed that LDCT screening can detect clinically significant cases of ILD in participants who fulfil criteria for commencing treatment. In addition, due to the size of this cohort, the current data provides a robust estimate of the clinical activity to be anticipated by ILD services as lung cancer screening rolls out across various geographical jurisdictions. Further research is needed to clarify findings in other large screening cohorts and to assess the long-term outcomes from screen-detected ILA and ILD.

10.1136/bmjresp-2022-001490.supp1 Supplementary data

## Data Availability

All data relevant to the study are included in the article or uploaded as supplementary information.
